# Newcastle disease virus RNA-induced IL-1β expression via the NLRP3/caspase-1 inflammasome

**DOI:** 10.1186/s13567-020-00774-0

**Published:** 2020-04-10

**Authors:** Pei Gao, Libin Chen, Lei Fan, Jinlian Ren, Haoyun Du, Minhua Sun, Yaling Li, Peng Xie, Qiuyan Lin, Ming Liao, Chenggang Xu, Zhangyong Ning, Chan Ding, Bin Xiang, Tao Ren

**Affiliations:** 1 College of Veterinary Medicine, South China Agricultural University, Guangzhou, 510642 China; 2 Key Laboratory of Animal Vaccine Development, Ministry of Agriculture, Guangzhou, China; 3 National and Regional Joint Engineering Laboratory for Medicament of Zoonosis Prevention and Control, Guangzhou, China; 4 Key Laboratory of Zoonosis Prevention and Control of Guangdong Province, Guangzhou, China; 5 Henan Institute of Science and Technology, Xinxiang, 453003 Henan China; 6 Shanghai Veterinary Research Institute, Chinese Academy of Agricultural Sciences, Shanghai, 200241 China

## Abstract

Newcastle disease virus (NDV) infection causes severe inflammation and is a highly contagious disease in poultry. Virulent NDV strains (GM) induce large quantities of interleukin-1β (IL-1β), which is the central mediator of the inflammatory reaction. Excessive expression of IL-1β exacerbates inflammatory damage. Therefore, exploring the mechanisms underlying NDV-induced IL-1β expression can aid in further understanding the pathogenesis of Newcastle disease. Here, we showed that anti-IL-1β neutralizing antibody treatment decreased body temperature and mortality following infection with virulent NDV. We further explored the primary molecules involved in NDV-induced IL-1β expression from the perspective of both the host and virus. This study showed that overexpression of NLRP3 resulted in increased IL-1β expression, whereas inhibition of NLRP3 or caspase-1 caused a significant reduction in IL-1β expression, indicating that the NLRP3/caspase-1 axis is involved in NDV-induced IL-1β expression. Moreover, ultraviolet-inactivated GM (chicken/Guangdong/GM/2014) NDV failed to induce the expression of IL-1β. We then collected virus from GM-infected cell culture supernatant using ultracentrifugation, extracted the viral RNA, and stimulated the cells further with GM RNA. The results revealed that RNA alone was capable of inducing IL-1β expression. Moreover, NLRP3/caspase-1 was involved in GM RNA-induced IL-1β expression. Thus, our study elucidated the critical role of IL-1β in the pathogenesis of Newcastle disease while also demonstrating that inhibition of IL-1β via anti-IL-1β neutralizing antibodies decreased the damage associated with NDV infection; furthermore, GM RNA induced IL-1β expression via NLRP3/caspase-1.

## Introduction

Newcastle disease (ND) is a highly contagious disease of poultry that leads to acute fever and sepsis and is caused by the Newcastle disease virus (NDV). This virus consists of a negative-sense, single-stranded RNA genome of approximately 15 190 nucleotides that encodes the structural proteins NP, P, M, F, HN, and L and the non-structural proteins V and W. Infection with virulent strains of NDV causes a strong immune response, with different organs expressing varying degrees of exudative inflammation, resulting in the release of large quantities of inflammatory factors, such as IL-1β, IL-6, IL-18, and IFN-β [1, 2]. Thus, exploring the mechanisms underlying the NDV-induced inflammatory response can aid in understanding the pathogenesis of Newcastle disease.

IL-1β is an essential component of the inflammatory process. As the central mediator of the inflammatory reaction, IL-1β increases the synthesis and release of IL-6, intercellular adhesion molecules, and vascular cell adhesion molecules, which activate lymphocytes and promote the infiltration of leukocytes and eosinophils into the site of inflammation [3]. An appropriate amount of IL-1β expression can repair damage and reduce viral proliferation. However, excessive IL-1β expression exacerbates the degree of inflammation, thereby increasing morbidity and mortality. Although elevated IL-1β expression is common with NDV infection, it is not unique to this virus, as several others, including influenza A virus, infectious bronchitis virus, Sendai virus, and vesicular stomatitis virus, also increase IL-1β expression [4–7]. In fact, a study on influenza A virus found that following inhibition of the IL-1β receptor, lung pathology caused by the influenza virus was significantly reduced, indicating that IL-1β is a principal component associated with pulmonary inflammation during H1N1 influenza viral infection [8]. Other studies have shown that neutralizing IL-1α and IL-1β with specific neutralizing antibodies effectively reduces respiratory inflammation induced by influenza A virus [9]. Furthermore, intraperitoneal injection of the IL-1Rα receptor or IL-1β monoclonal antibody in mice effectively reduces the degree of inflammation induced by *Propionibacterium acnes* [10]; however, IL-1β knockdown in animals showed increased pathogenesis and lethality of Sindbis virus infection [11]. Animals that lack the IL-1β receptor exhibit increased susceptibility to the West Nile virus [12]. Thus, it remains unclear whether the IL-1β pathway is beneficial or harmful for hosts during viral infection. Currently, it is known that NDV induces the expression of IL-1β, but whether this expression is excessive or causes inflammatory damage remains unknown.

Viruses induce the expression of IL-1β via the nucleotide binding of the oligomerization-domain leucine-rich repeats containing the pyrin domain 3 (NLRP3) inflammasome [13, 14]. The NLRP3 inflammasome consists of NLRP3, apoptosis-associated speck-like proteins containing a CARD (ASC), and caspase-1. Activation of this inflammasome requires two signals. The first is a pre-stimulatory signal, which is activated by the NF-κB signalling pathway and promotes the expression of NLRP3. The second is referred to as the activation signal, which alters the structure of NLRP3 after oligomerization, recruits ASC, activates caspase-1, and cleaves IL-1β precursors to form mature IL-1β [15, 16]. Moreover, this process is essential for the expression of pro-inflammatory cytokines, the regulation of inflammation, and innate immune responses [17]. Numerous factors activate the NLRP3 inflammasome, including ATP, alum, ultraviolet radiation, and muramyl dipeptide, as well as RNA or DNA from viral, bacterial, and fungal pathogens [18]. DNA viruses, such as poxvirus, herpes simplex virus, and adenovirus, can activate NLRP3 inflammasomes and promote IL-1β expression [19, 20], while myxoma virus activates the NLRP3 inflammasome by inducing ROS and cathepsin B [21, 22]. Many RNA viruses, such as infectious bronchitis virus, porcine reproductive and respiratory syndrome virus, and swine fever virus, induce IL-1β expression via the NLRP3 inflammasome [23–25]. The introduction of NLRP3 into ducks using a lentiviral expression system significantly increased the expression of NLRP3, IL-1β and IL-18 induced by *E. coli* and enhanced the degree of inflammation throughout the body [26]. The use of the NLRP3 inhibitor MCC950, the caspase-1 inhibitor Ac-YVAD-CHO and IL-1β neutralizing antibodies effectively reduce the asthma-induced inflammatory response [27], indicating that activation of the NLRP3 inflammasome and induction of IL-1β are important components in inflammation in the body. NLRP3 activation and caspase-1 cleavage are essential for the maturation and expression of IL-1β. NDV activates the NLRP3 inflammasome and increases the secretion of IL-1β in mice and human macrophages [4]. However, in poultry, which are the natural hosts of NDV, NLRP3 inflammasome-mediated expression of IL-1β is still unclear.

Viruses invade host cells and utilize the host nucleic acids and proteins to induce the synthesis of viral components to produce new virions. Various structural components, such as viral nucleic acids, ion channel proteins, and non-structural proteins produced during the replication process, can activate the expression of IL-1β [28]. Following influenza virus infection, the host cell recognizes viral genomic RNA through TLR7 and increases IL-1β expression, which is dependent upon activation of NLRP3/caspase-1 inflammasomes [5]. Arteritis viral RNA binds to DDX19A, which helps activate NLRP3 and promotes IL-1β expression [29]. Stimulation of THP1 cells by the hepatitis C virus RNA genome causes IL-1β mRNA levels to increase significantly, which is dependent on the NLRP3 inflammasome complex [30]. 3D proteins of enterovirus 71 also activate NLRP3 inflammasomes and promote the expression of IL-1β [31]. Non-structural proteins of some viruses can also promote IL-1β production. For example, the PB1-F2 and M2 proteins of influenza virus activate the NLRP3 inflammasome and promote the maturation of IL-1β [32, 33]. Furthermore, the 2B viroporin of encephalomyocarditis virus, the 2B protein of rhinovirus, and the E proteins of severe acute respiratory syndrome coronavirus also induce the expression of IL-1β [34–36]. Consequently, induction of IL-1β expression by viruses is likely mediated by different viral components. However, the specific elements of NDV that mediate IL-1β expression during NDV infection have not yet been determined.

In the present study, the effect of IL-1β during infection with virulent NDV was investigated utilizing IL-1β-specific neutralizing antibodies. The ability of NDV to activate NLRP3 inflammasome reactions in poultry was also confirmed. Finally, the primary components of NDV that activate the NLRP3 inflammasome complex and promote IL-1β production were evaluated in an effort to clarify the molecular mechanisms involved in NDV-induced IL-1β expression.

## Materials and methods

### Virus, cells, and animals

The virulent GM NDV strain (chicken/Guangdong/GM/2014), which was isolated from dead chickens in China’s Guangdong Province [37, 38], and the lentogenic vaccine strain La Sota were obtained from stocks maintained in our laboratory. Viruses were propagated in 9-day-old specific pathogen-free (SPF) embryonated chicken eggs, and the allantoic fluid was centrifuged, subpackaged, and stored at −80 °C until use. The viral median egg infectious dose (EID_50_) was determined in SPF embryonated eggs. The median tissue culture infective dose (TCID_50_) of viruses was detected in DF1 cells using the Reed and Muench method. Preparation of ultraviolet (UV)-inactivated NDV occurred as follows: GM virus was dispersed in a cell culture dish, and then a UV lamp was placed above the dish for 5 hours. DF1 cells, which were maintained in our laboratory, were cultured in Dulbecco’s modified Eagle’s medium (DMEM; Gibco, Shanghai, China) with 10% foetal bovine serum (FBS; Gibco) at 37 °C and 5% CO_2_. For in vivo studies, 8-week-old healthy SPF chickens were purchased from Guangdong Wens Dahuanong Biotechnology Co., Ltd. (Guangdong Wens Dahuanong Biotechnology Co., Ltd, Yunfu, China) and housed in micro-isolator cages. Three-month-old New Zealand white rabbits were provided by the Guangdong Medical Laboratory Animal Centre.

### Animal experiments

*Experiment 1*: Thirty-nine healthy 8-week-old SPF chickens were randomized into three groups (*n* = 13). All birds in one group were inoculated intranasally (i.n.) with 10^5^ EID_50_ of GM strain NDV in a volume of 200 μL per bird, and chickens in the other group were inoculated with an equal volume of phosphate buffered saline (PBS, PH = 7.4). At three days post-inoculation (dpi), 3 inoculated chickens in each group were euthanized, and the lungs, glandular stomach, and bursa of Fabricius were harvested and frozen at −80 °C for subsequent testing. The ten remaining chickens were observed for clinical signs such as morbidity and mortality. The temperature of each chicken was measured once per day.

*Experiment 2*: To prepare the rabbit anti-IL-1β neutralizing antibody, the chicken *IL*-*1β* gene sequence (GenBank accession no. HM179638.1) was amplified by PCR using chicken lung tissue samples. The forward and reverse primers were 5ʹ-GAATTCATGGCGTTCGTTCCCGACC-3ʹ and 5ʹ-CTCGAGGCGCCCACTTAGCTTGTAG-3ʹ, respectively. The amplified *IL*-*1β* gene was cloned into the vector PET-32a (Novagen, EMD-Millipore, Billerica, MA, USA) and expressed in *E. coli* strain BL21(DE3). After confirmation by Western blot with anti-His antibodies (Tiangen, Beijing, China), the synthesized protein was purified using Ni–NTA on a Ni-chelating column. Two New Zealand white rabbits were subcutaneous injection (s.c.). with 1 mg of purified IL-1β emulsified with complete Freund’s adjuvant, with the corresponding blank controls. At 14, 21, and 28 days after the first injection, the rabbits were injected s.c. with 1 mg of IL-1β emulsified with incomplete Freund’s adjuvant. Two weeks after the final boost, rabbit serum containing neutralizing antibodies was collected. We also constructed a eukaryotic expression plasmid, pCAGGS-IL-1β, and transfected it into DF1 cells. Cellular proteins were collected at 48 h after transfection. The specificity of the antibody against the cellular protein chicken IL-1β was determined by Western blot. The chicken IL-1β protein was detected using the 1:500 diluted IL-1β neutralizing primary antibody.

Forty healthy 8-week-old SPF chickens were randomized into four groups (*n* = 10). All birds in the GM + anti-IL-1β, GM + negative serum and GM groups were inoculated i.n. with 10^5^ EID_50_ of GM strain NDV in a volume of 200 μL per bird, and chickens in the control group were inoculated with an equal volume of PBS. At 1 dpi, the chickens were treated with 150 μL of anti-IL-1β neutralizing antibody in the GM + anti-IL-1β group, negative serum in the GM + negative serum group or PBS in the GM and control groups via intraperitoneal injection (i.p.). Subsequent injections were performed once a day for 4 days. At 3 dpi, three chickens in each group were euthanized, the lungs and glandular stomach were harvested from each bird and frozen at −80 °C for subsequent testing, and the seven remaining chickens were observed for morbidity and mortality. The temperature of each chicken was measured once per day.

### Immunohistochemical detection

Organ samples from the chickens were collected and fixed with 4% paraformaldehyde. Immunohistochemical detection was performed according to the methods provided in the literature [39]. Rabbit anti-IL-1β polyclonal antibody was utilized for immunohistochemical staining of NDV-infected tissues, as well as tissues from the IL-1β neutralizing antibody- or negative serum-treated group and the control group of uninfected normal chickens. Primary antibody binding was detected via anti-rabbit-HRP (Zhongshan Golden Bridge, Beijing, China).

### Viral infection and chemical treatment assay

DF1 cells were seeded 16 h prior to infection in six-well plates at a density of 2 × 10^5^ cells/well. The cells were washed three times with PBS and then infected with the NDV strains GM or La Sota at a multiplicity of infection (MOI) of 1.0. Following a 1 h absorption period at 37 °C, unattached virus as removed, and the cells were then washed three times with PBS and cultured in DMEM at 37 °C. Trypsin at a concentration of 1 μg/mL was added to the DMEM in which the La Sota virus was cultured. Culture supernatants and cells were harvested at 6, 12, 24, 36, and 48 h for further studies.

For chemical treatment experiments, DF-1 cells were pretreated with 20 μM Ac-YVAD-CHO (Sigma, St Louis, MO, USA) or DMSO (Sigma, St. Louis, MO, USA) for 1 h, and then the cells were incubated with GM NDV at a MOI of 1, together with each inhibitor, in a 5% CO_2_ incubator at 37 °C for 24 h. Culture supernatants and cells were harvested for further studies.

### Transfection of small interfering RNA

NLRP3-specific silencing RNA (sense: 5′-CCUGGAGGGCAAGCAUUAUTT-3′; antisense: 5′-AUAAUGCUUGCCCUCCAGGTT-3′) was designed and synthesized by GenePharma (GenePharma Co., Ltd., Suzhou, China). DF-1 cells were grown to 70% confluence in 60-mm cell culture dishes and transfected with 4 μL scrambled control (NC) Si-RNA (20 nM) or NLRP3-specific Si-RNA (20 nM) with Lipofectamine 2000 (Invitrogen, Carlsbad, CA, USA). At 24 h post-transfection, cell culture supernatants were removed, and the cells were infected with GM NDV at a MOI of 1 for 24 h. Cell culture supernatants and cell lysates were harvested for enzyme-linked immunosorbent assay (ELISA) and Western blot analysis, respectively.

### Cell viability assay

The viability of DF1 cells was measured using a CCK-8 cell proliferation and cytotoxicity assay kit (Abmole Bioscience, Houston, TX, USA). Briefly, 5 × 10^3^ DF1 cells were cultured in a 96-well plate. After transfection with Si-NLRP3 or treatment with AcYVAD-CHO, 10 μL CCK-8 was added to each well, and the cells were incubated at 37 °C for 2 h. Then, the absorbance was measured at a wavelength of 450 nm (and a reference wavelength at 690 nm) against the background control using a microplate reader.

### Viral RNA isolation and transfection

DF1 cells were infected with GM NDV at a MOI of 1, and the cells were incubated with the same volume of DMEM as the mock control. When the GM NDV-infected cells showed maximal cytopathic effects, all cells were frozen at −80 °C and freeze-thawed three times. The supernatants of the mock control and GM-infected cells were collected and centrifuged at 5000 *g* for 30 min at 4 °C and passed through 0.45 mm filters. The filtered supernatants were centrifuged at 35 000 rpm for 2 h, and NDV virions at the bottom of the centrifuge tube were collected [29]. The E.Z.N.A. viral RNA kit (Omega, GA, USA) was used to extract RNA. DF1 cells were transfected with RNA from the GM NDV strain with Lipofectamine 2000, and a corresponding negative control was performed. At the indicated time points, cell culture supernatants were harvested for the detection of IL-1β.

### Quantitative real-time polymerase chain reaction (qRT-PCR)

Extracted RNA (1 μg) was reverse transcribed to cDNA by utilizing ReverTra Ace qPCR RT master mix with gDNA remover (Toyobo, Japan). cDNA was subjected to qRT-PCR using SYBR qPCR mix (Toyobo, Japan). We designed primers (Table 1) used for qRT-PCR based on previously reported target sequences [40]. qRT-PCR was performed in a 7500 Fast Real-Time PCR system according to previously reported methods [41].

**Table 1:** qPCR primers utilized in this study.

Primer names	Sequence (5′–3′)	GenBank no.
IL-1β-F	CTGGGCATCAAGGGCTACAA	AB559570.2
IL-1β-R	CGGTAGAAGATGAAGCGGGT	
NDV-F	AGTGATGTGCTCGGACCTTC	DQ486859
NDV-R	CCTGAGGAGGCATTTGCTA	
GAPDH-F	CCTCTCTGGCAAAGTCCAAG	V00407
GAPDH-R	CATCTGCCCATTTGATGTTG	

### Western blot analysis

DF1 cells were lysed with RIPA buffer supplemented with PMSF and phosphorylase inhibitors (Beyotime, Shanghai, China), and the protein concentration was tested with a Bradford assay (Bio-Rad, Hercules, CA, USA). Denatured protein (30 μg) was electrophoresed in a 10% sodium dodecyl sulfate–polyacrylamide gel electrophoresis (SDS-PAGE) gel and transferred to an NC membrane (GE, MA, USA). NC membranes were blocked and incubated with specific rabbit anti-NLRP3 (provided by Professor Zhangyong Ning of South China Agricultural University), anti-NDV-NP antibody, or rabbit anti-GAPDH antibody (Cat: ab181602, Abcam, UK) for 12 h at 4 °C. The primary antibody was removed, and the membrane was incubated with IRDye 800-conjugated anti-rabbit IgG (Cat: 926-32211, LI-COR Biosciences, Lincoln, NE, USA) for 45 min at 25 °C–30 °C. The membranes were visualized using an Odyssey infrared imaging system (LI-COR Biosciences, Lincoln, NE, USA).

### ELISA

At the indicated time points post-infection, cell culture supernatants were harvested and centrifuged at 5000 rpm for 25 min at 4 °C. A chicken IL-1β ELISA kit (Cat: SEA563Ga, USCN Sciences Co. Ltd., Wuhan, China) was used to determine the concentration of IL-1β.

### Caspase-1 activity assay

The activity of caspase-1 in DF-1 cells was measured using a caspase-1 activity assay kit (Cat: C1102, Beyotime, Shanghai, China). Cells were digested with trypsin, centrifuged with cell culture supernatants at 1000 rpm for 5 min at 4 °C, and washed once with PBS. Then, the cell pellet was collected, lysed, and centrifuged at 20 000 rpm for 20 min at 4 °C, and the activity of caspase-1 was determined based on the ability of caspase-1 to convert acetyl-Tyr-Val-Ala-Asp p-nitroaniline (Ac-YVAD-pNA) into p-nitroaniline (pNA).

### Statistical analysis

Statistical analysis and data presentation were performed using GraphPad Prism software version 5.0 (GraphPad Software, Inc., La Jolla, CA, USA). A standard two-tailed unpaired Student’s *t*-test was used to calculate *p* values, which was considered significant if ≤ 0.05. The sample sizes, specific statistical tests used, and the main effects of our statistical analyses for each experiment are detailed in each figure legend.

## Results

### NDV induces IL-1β expression in vivo and in vitro

**Figure 1:**
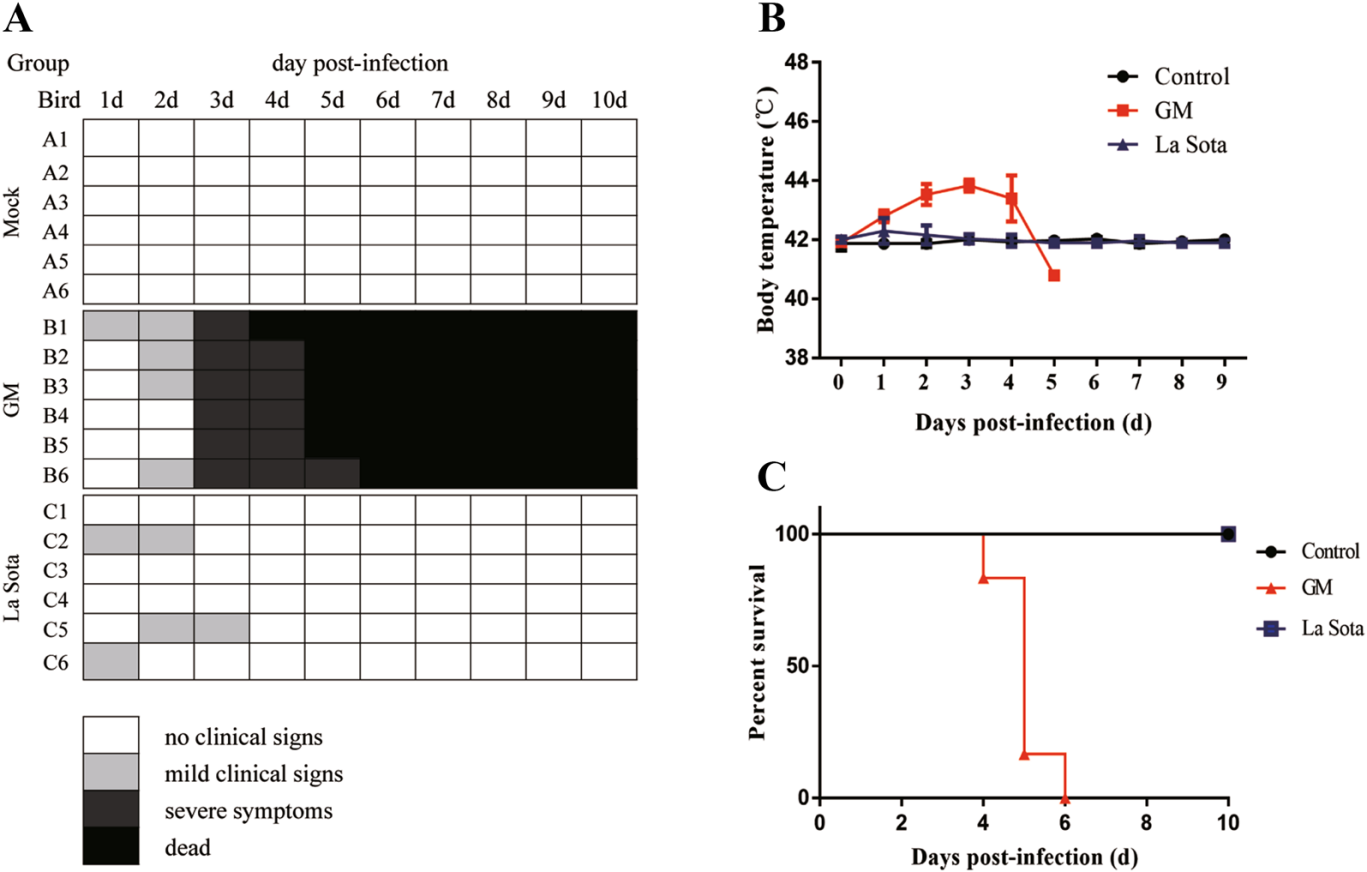
**Clinical signs, temperature, and mortality of 8-week-old SPF chickens following NDV viral challenge.A** Clinical signs were assessed once daily, and symptoms were scored according to severity. **B** Temperature changes in the chickens was measured once daily. **C** Mortality of SPF chickens infected with NDV or PBS. Ten chickens were observed for survival over 10 days. The data are expressed as percent total survival.

To determine whether NDV induces IL-1β expression in vivo, we infected 8-week-old SPF chickens with 10^5^ EID_50_ i.n. with either the genotype VII virulent GM NDV strain, the lentogenic La Sota strain, or PBS as a control. Chickens that were infected with La Sota expressed mild clinical symptoms (Figure 1A), and their temperatures returned to normal after a slight increase at 1 dpi (Figure 1B); no deaths occurred in this group (Figure 1C). Conversely, following infection with the virulent GM NDV strain, the animals showed depression, anorexia, and neurological symptoms at 3 dpi (Figure 1A). Their temperatures began to increase at 1 dpi, peaked (44 °C) at 3 dpi and were significantly higher than the temperatures observed in the control group (Figure 1B). The mortality rate was 100% (Figure 1C), denoting that infection with virulent strains of NDV causes greater morbidity and mortality than that of less virulent strains.

**Figure 2:**
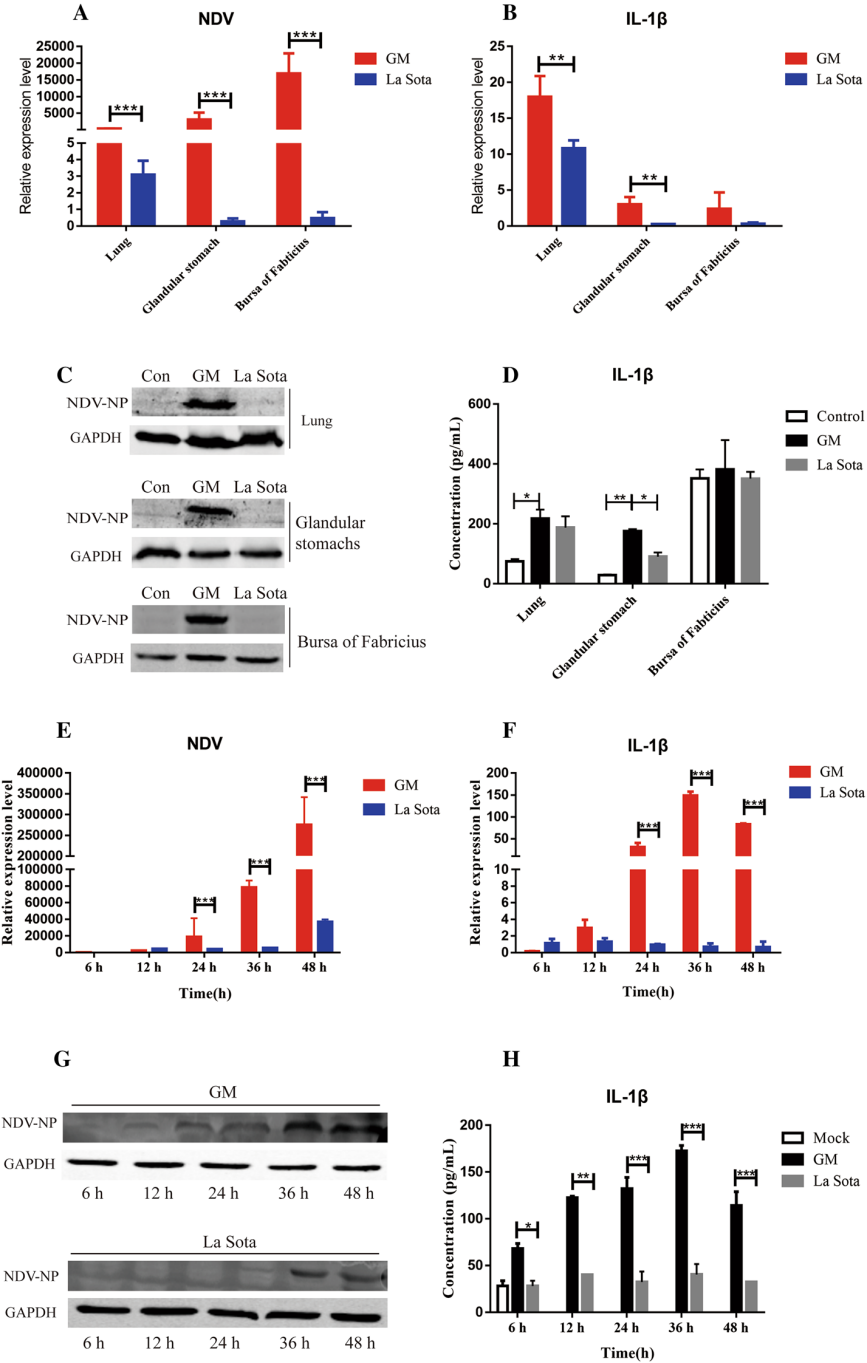
**NDV and IL-1β expression levels in the organs of SPF chickens and DF1 cells.A** NDV M gene expression in the lungs, glandular stomach, and bursa of Fabricius of chickens. **B** IL-1β gene expression in the lungs, glandular stomach, and bursa of Fabricius of chickens. **C** NDV-NP protein expression in the lungs, glandular stomach, and bursa of Fabricius in chickens. **D** IL-1β protein expression in the lungs, glandular stomach, and bursa of Fabricius of chickens. The lungs, glandular stomach, and bursa of Fabricius were collected at 3 dpi, and levels of NDV and IL-1β were determined by qPCR, Western blot, or ELISA. **E** NDV M gene expression in DF1 cells. **F** IL-1β gene expression in DF1 cells. **G** NDV-NP protein expression in DF1 cells. **H** IL-1β protein expression in DF1 cells. DF1 cells and cell culture supernatant were collected at the indicated time points, and the levels of NDV and IL-1β were determined by qPCR, Western blot, or ELISA. *P < 0.05, **P < 0.01, and ***P < 0.001 between the experimental and control groups.

Expression levels of the NDV M gene and IL-1β were determined at 3 dpi. Following infection with the GM strain, NDV RNA expression was upregulated in the lungs, glandular stomach, and bursa of Fabricius, whereas La Sota-infected chickens showed significant proliferation in the lungs but not in the glandular stomach or bursa of Fabricius (Figure 2A). The gene expression levels of IL-1β in chickens that were infected with the GM or La Sota strains increased by 17.9- or 10.8-fold in the lungs, 3.1- or 0.3-fold in the glandular stomach, and 1.3- or 0.3-fold in the bursa of Fabricius, respectively, compared with those in the control group (Figure 2B). Additionally, the protein expression levels of NDV-NP increased with time (Figure 2C), and IL-1β increased 1.5- or 1.3-fold in the lungs, 6.1- or 3.2-fold in the glandular stomach, and 1.1-fold or not at all in the bursa of Fabricius of GM- or La Sota-infected chickens, respectively, compared to the levels in the control chickens (Figure 2D). Thus, IL-1β expression was found in all of the chickens in this study, but the highest level of expression was induced by the virulent NDV strain.

In vitro, laboratory testing sought to discover whether NDV promotes increased levels of IL-1β expression outside of the host chickens. Thus, the protein expression of cytokines was measured in the laboratory via NDV infection of DF1 cells. The results clearly showed that the expression of the NDV M gene and NP protein gradually increased over time (Figures 2E and G). Specifically, the IL-1β gene levels in the DF1 cells increased gradually following GM infection, reaching a final peak (148.5-fold) at 36 h post-infection (hpi) (Figure 2F). IL-1β protein levels in DF1 cells also increased gradually post GM infection, reaching the final peak (172.7 pg/mL) at 36 hpi (Figure 2H). IL-1β levels were significantly higher than all of the samples in the control group (Figures 2F and H). In summary, NDV induced high levels of IL-1β expression in vivo as well as in vitro.

### Anti-IL-1β neutralizing antibodies reduce the pathology of virulent NDV infection

**Figure 3:**
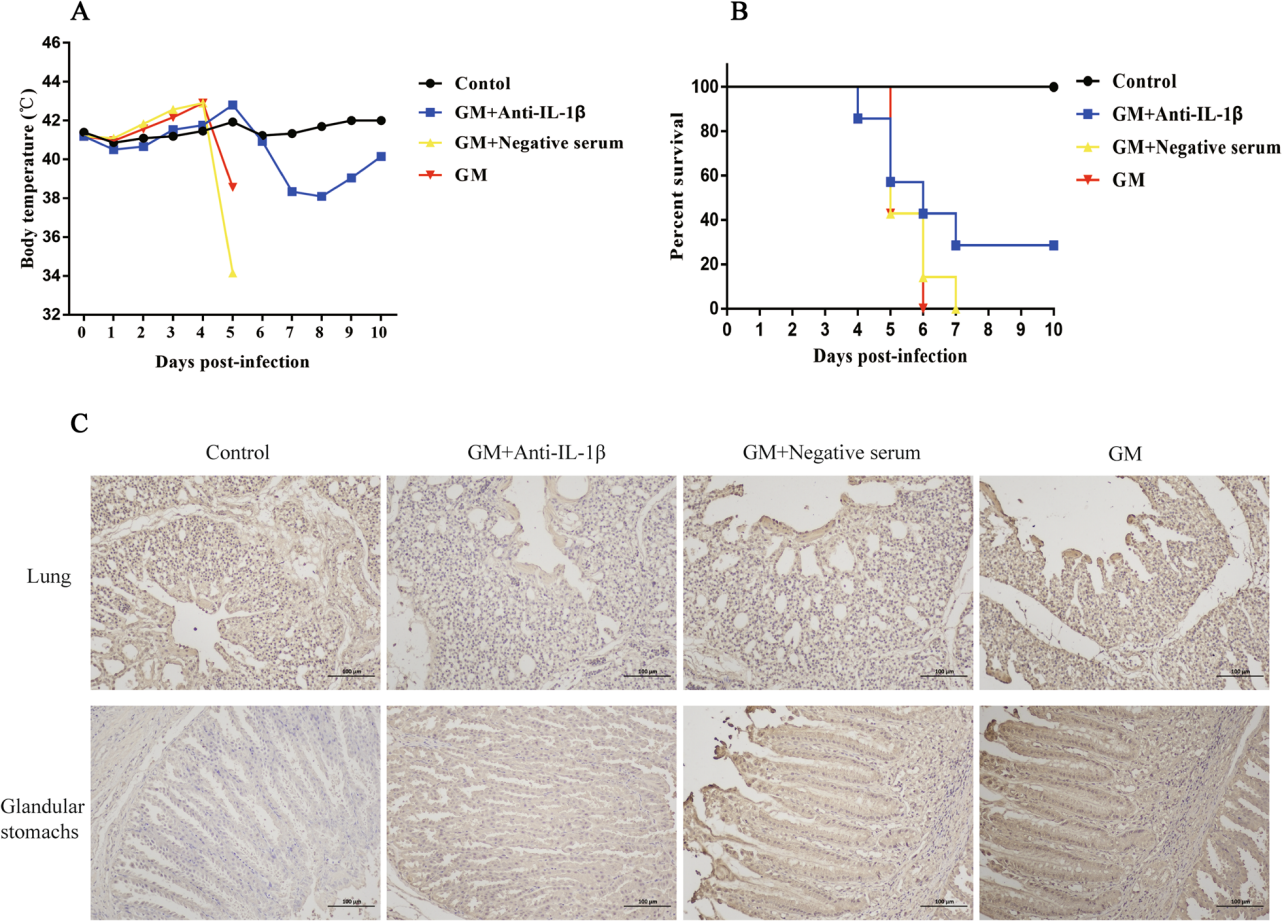
**Temperature, mortality, and gene expression in NDV-infected chickens after anti-IL-1β neutralizing antibody treatment.A** Temperature change in the chickens. **B** Mortality of SPF chickens after NDV infection and anti-IL-1β neutralizing antibody treatment. **C** Immunohistochemical detection of IL-1β in the lungs and glandular stomach of chickens. Seven chickens were observed for survival over 10 days. The data are expressed as percent total survival. Temperature was measured once daily.

This experiment evaluated whether increased expression of IL-1β during NDV infection was associated with high morbidity and mortality. For this part of the study, we first prepared a neutralizing antibody against chicken IL-1β and verified the antibody using Western blot (Additional file 1). The results demonstrated that the prepared antibody was specific for chicken IL-1β. Then, eight-week-old SPF chickens were administered GM NDV and treated with either anti-IL-1β neutralizing antibody, negative serum, or PBS as the negative controls. The body temperature of each chicken and the deaths of the chickens were recorded daily. The results showed that treatment with anti-IL-1β neutralizing antibody decreased body temperature compared to that after treatment with negative serum or PBS after GM infection (Figure 3A). The body temperatures of the two chickens in the anti-IL-1β-treated group gradually increased after infection, peaked on the 5^th^ day, rapidly decreased, and then slowly returned to normal (Figure 3A). Overall, IL-1β protein expression in anti-IL-1β neutralizing antibody-treated chickens was lower than that in the negative serum-treated chickens or the negative control group (Figure 3C). More importantly, the mortality of the anti-IL-1β-treated chickens decreased from 100% to 71%, and these results were compared to those of chickens treated with negative serum or PBS (Figure 3B). These results clearly indicated that neutralization of IL-1β decreased both the pathology and mortality induced by virulent NDV.

### NDV induces IL-1β expression via NLRP3/caspase-1 inflammasomes

**Figure 4:**
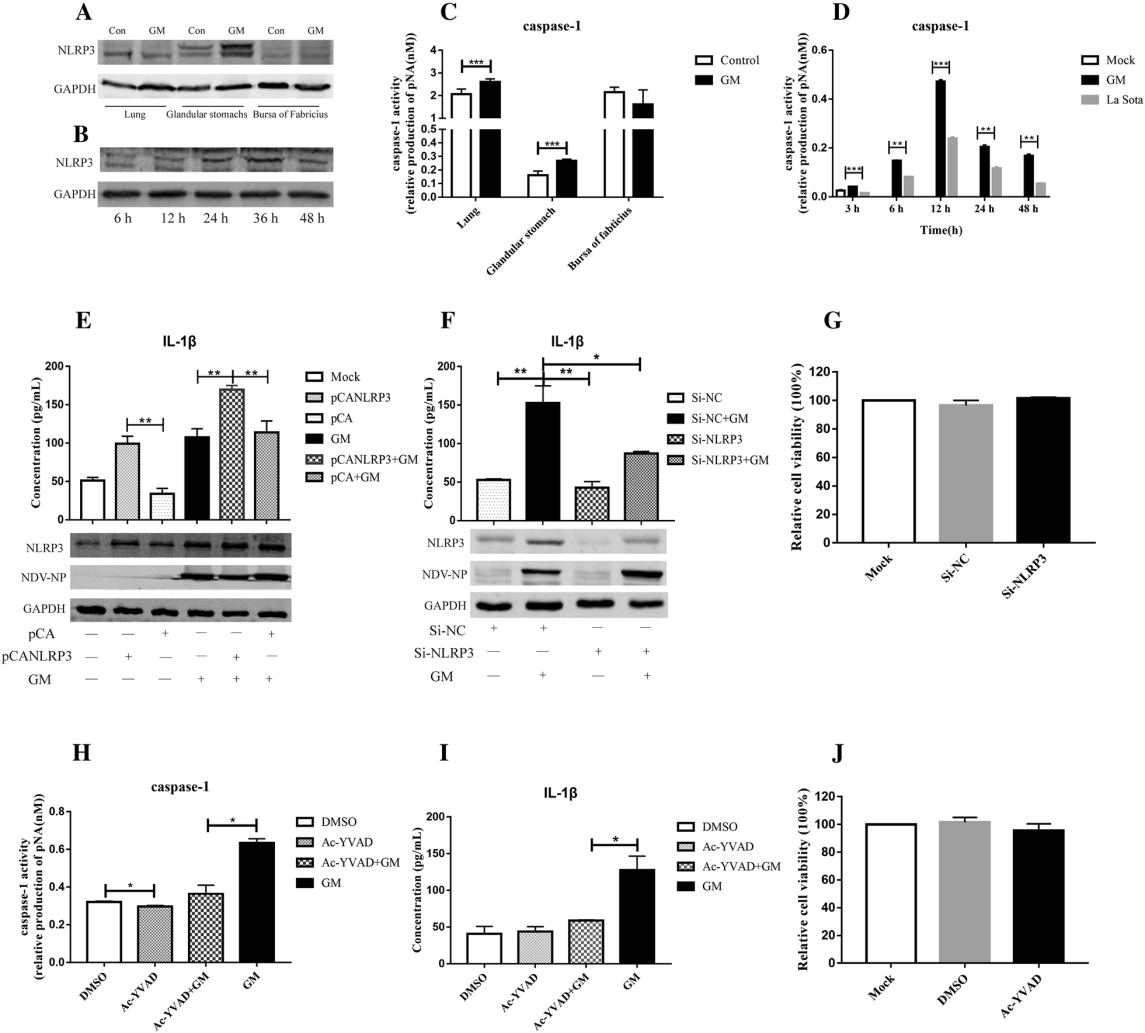
**NDV induction of IL-1β expression through NLRP3/caspase-1.A** NLRP3 expression in the lungs, glandular stomach, and bursa of Fabricius of chickens. **B** NLRP3 expression in DF1 cells. **C** Activation of caspase-1 in the lungs, glandular stomach, and bursa of Fabricius of chickens. **D** Activation of caspase-1 in DF1 cells. DF1 cells and the organs of chickens at 3 dpi were collected for detection of caspase-1 activity using a caspase-1 activity assay kit. **E** NLRP3 and IL-1β expression after overexpression of NLRP3 and infection with GM NDV. **F** NLRP3 and IL-1β expression after transfection with i-NLRP3 and infection with GM NDV. **G** The viability of DF1 cells that were transfected with Si-NC or Si-NLRP3 was measured by CCK-8 cell proliferation and cytotoxicity assay kits. DF1 cells were transfected with an NLRP3 overexpression plasmid or Si-NLRP3 for 24 h and then infected with GM NDV at a MOI of 1 for 24 h. Cells and cell culture supernatant were collected, and the expression of NLRP3 and IL-1β was assessed by Western blot and ELISA. **H** Caspase-1 activation after incubation with the caspase-1-specific inhibitor Ac-YVAD-CHO. **I** IL-1β expression after incubation with the caspase-1-specific inhibitor Ac-YVAD-CHO. **J** The viability of DF1 cells that were treated with Ac-YVAD-CHO was measured by CCK-8 cell proliferation and cytotoxicity assay kits. DF1 cells were incubated with Ac-YVAD-CHO (20 μM) for 1 h and then infected with GM NDV at a MOI of 1 for 24 h. Cells and cell culture supernatant were collected at 24 hpi, and caspase-1 activity and IL-1β expression were assessed. *P < 0.05, **P < 0.01, and ***P < 0.001 between the experimental and control groups.

To determine how NDV induces IL-1β expression in poultry, the role of the NLRP3/caspase-1 inflammasome was investigated. NDV has been reported to induce IL-1β expression by activating the NLRP3 inflammasome in mammalian cells [4]. However, in poultry, whether NDV induces IL-1β expression via NLRP3/caspase-1 is still unclear. This study found that NDV induced the expression of NLRP3 in the glandular stomach (Figure 4A). The expression of NLRP3 increased with time in DF1 cells after GM infection (Figure 4B). NDV also induced the activation of caspase-1 in the organs (Figure 4C), and the activation of caspase-1 increased with time in DF1 cells (Figure 4D). After overexpression of NLRP3 and infection with GM, IL-1β expression increased from 107.5 pg/mL to 169.3 pg/mL (Figure 4E), and after inhibition of NLRP3 with Si-RNA, the expression of IL-1β decreased from 152.8 pg/mL to 87.0 pg/mL (Figure 4F). When the activity of caspase-1 was inhibited by Ac-YVAD-CHO (Figure 4H), IL-1β expression decreased from 127.5 pg/mL to 58.9 pg/mL (Figure 4I). Cell viability was unaffected by Si-RNA or Ac-YVAD-CHO treatment (Figures 4G and J). These results indicated that the NLRP3/caspase-1 inflammasome is associated with the NDV-induced expression of IL-1β in poultry.

### NDV RNA induces IL-1β expression

**Figure 5:**
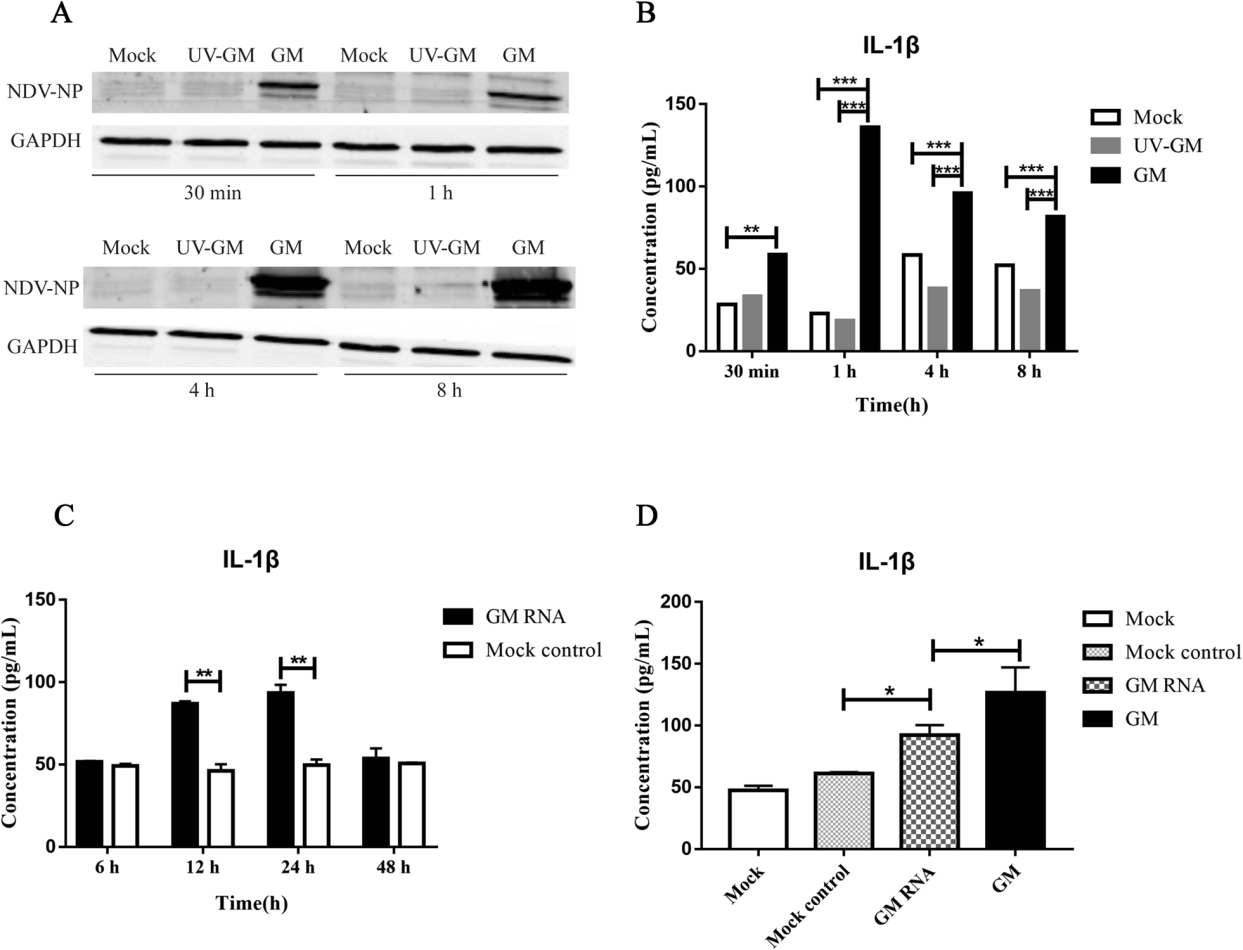
**NDV RNA induction of IL-1β expression.A** NDV-NP expression after infection with UV-GM and GM NDV strains. **B** IL-1β expression after infection with UV-GM and GM NDV strains. **C** GM RNA induced the expression of IL-1β at various time points. **D** GM RNA and GM NDV induced IL-1β expression at 24 hpi. Cells were transfected with 2 μg GM RNA or infected with GM NDV at a MOI of 1. Samples were collected at the indicated time points for subsequent testing. *P < 0.05, **P <0.01, and ***P < 0.001 between the experimental and control groups.

To identify the exact NDV viral components that induce IL-1β expression, DF1 cells were infected with UV-inactivated GM NDV, and a new GM challenge group and negative control group were created. The results showed that NDV-NP increased with time after GM infection, but UV-GM failed to induce NDV-NP expression (Figure 5A), which indicated that UV-GM failed to proliferate effectively. The GM strain induced IL-1β expression; however, that same strain did not induce IL-1β expression following UV inactivation (Figure 5B). These results indicate that the induction of IL-1β by NDV might be related to NDV genomic RNA or the non-structural proteins V and W, which are produced during viral proliferation.

To examine the effects of NDV RNA on IL-1β expression, the virus was cultured with DF1 cells; ultracentrifugation was used to extract the viral supernatant from the culture, viral RNA was extracted and transfected into DF1 cells, and a corresponding negative control was set up. IL-1β protein expression levels were measured by ELISA. The results showed that with an increase in stimulation time, IL-1β expression in the GM RNA group increased gradually, reaching its peak (93.5 pg/mL) at 24 h and then decreasing gradually, whereas the expression of IL-1β in the control group did not increase significantly (Figure 5C). Unlike in the control group, both GM RNA and GM NDV induced IL-1β expression at 24 hpi; GM RNA-induced IL-1β expression was slightly less than that induced by GM NDV (Figure 5D). The above results indicate that GM RNA induces IL-1β production.

### GM RNA induces IL-1β expression via the NLRP3/caspase-1 inflammasome

**Figure 6:**
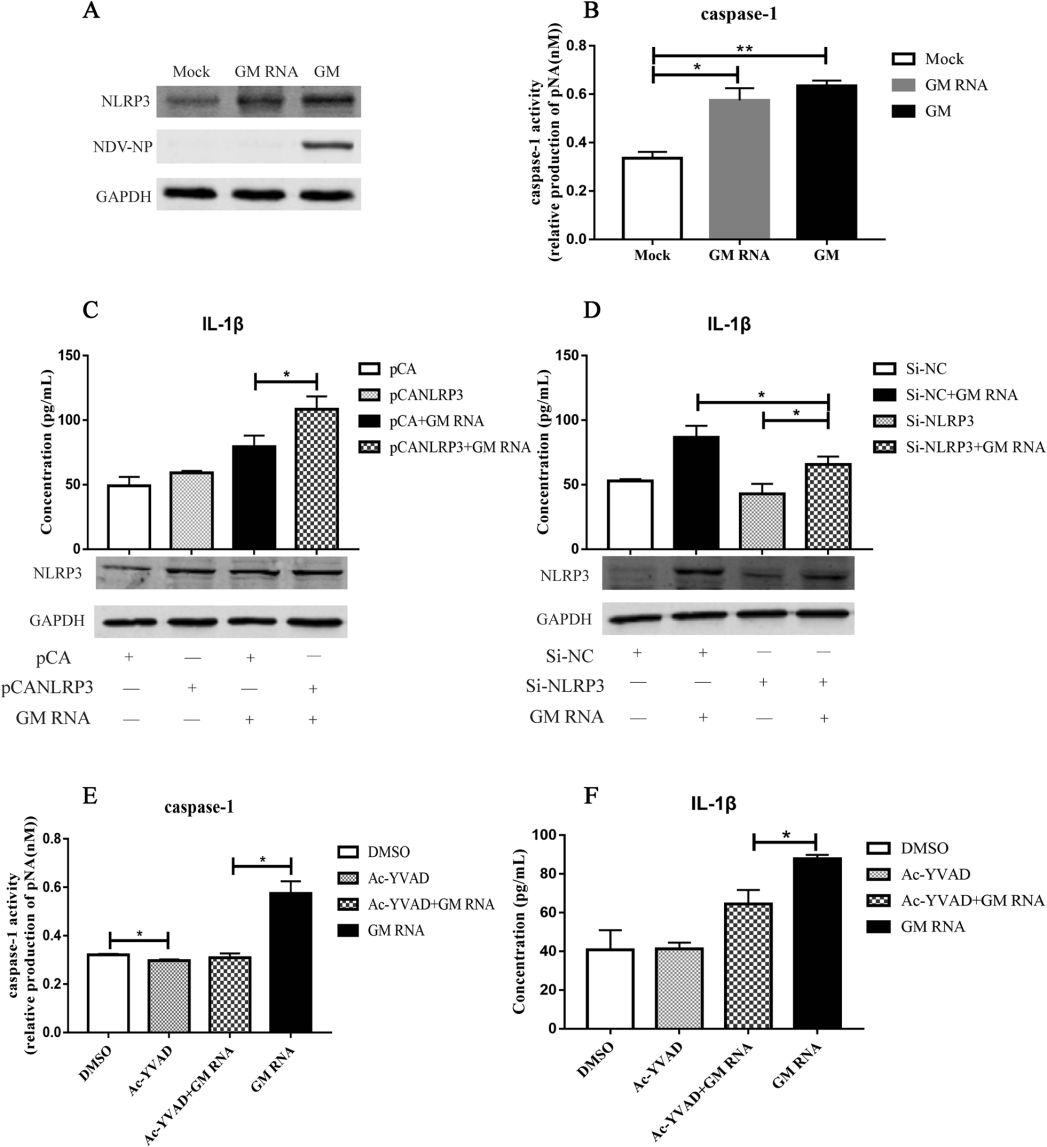
**GM RNA induction of IL-1β expression via NLRP3/caspase-1 inflammasomes.A** Activation of NLRP3 after GM RNA transfection and GM NDV infection. **B** Activation of caspase-1 after GM RNA transfection and GM NDV infection. **C** NLRP3 and IL-1β expression after overexpression of NLRP3 and stimulation with GM RNA. **D** NLRP3 and IL-1β expression after transfection with Si-NLRP3 and stimulation with GM RNA. DF1 cells were transfected with the NLRP3 overexpression plasmid or Si-NLRP3 for 24 h and then stimulated with GM RNA for 24 h. Samples were collected for subsequent testing. **E** Caspase-1 activation after incubation with the caspase-1-specific inhibitor Ac-YVAD-CHO and transfection with GM RNA. **F** IL-1β expression after incubation with the caspase-1-specific inhibitor Ac-YVAD-CHO and transfection with GM RNA. DF1 cells were incubated with Ac-YVAD-CHO (20 μM) for 1 h and then transfected with GM RNA for 24 h. Samples were collected for subsequent testing. *P < 0.05, **P < 0.01 between the experimental and control groups.of illness, the expression levels of IL-1β.

To confirm the signalling pathway involved in GM RNA-induced IL-1β expression, the activation of NLRP3 and caspase-1 following GM RNA transfection was examined. Western blot results showed that NLRP3 expression was significantly increased after stimulation with GM RNA and GM NDV (Figure 6A). Following GM RNA and GM stimulation, the activities of caspase-1 were 0.54 and 0.62, respectively, which were significantly higher than those in the mock control (Figure 6B). These results indicate that GM RNA activates the NLRP3/caspase-1 inflammasome.

Further studies found that after overexpression of NLRP3 and stimulation with GM RNA, IL-1β expression increased from 79.3 pg/mL to 108.5 pg/mL (Figure 6C), and after inhibition of NLRP3 by Si-RNA and stimulation with GM RNA, IL-1β expression induced by GM decreased from 86.5 pg/mL to 65.1 pg/mL (Figure 6D). When the activity of caspase-1 was inhibited by Ac-YVAD-CHO for 1 h and cells were stimulated with GM RNA for 24 h, the activity of caspase-1 was significantly decreased (Figure 6E). IL-1β expression decreased from 97.9 pg/mL to 64.6 pg/mL (Figure 6F), indicating that the NLRP3/caspase-1 inflammasome is involved in IL-1β expression induced by GM RNA.

## Discussion

NDV is one of the most important infectious diseases endangering the development of China’s poultry industry today. Chickens are extremely susceptible to this illness, and infection often results in mortality. Alternatively, other animals generally experience milder symptoms than chickens. For example, chickens may suffer serious, life-threatening clinical symptoms following infection with NDV, while other poultry, such as ducks exposed to the same virus, may experience only mild symptoms [37, 42]. This phenomenon may be due to the level of immune response generated by each species, which is mediated by cytokines [43]. In addition, significant differences were observed in the expression of inflammatory cytokines induced by NDV infection with strains that varied in virulence or genotype. For instance, chickens that were infected with genotype VIII NDV were reported to express significantly higher levels of IL-1β, IFN-γ, and IL-12α on the third day of infection than chickens that were infected with the genotype VII strain. Conversely, on the 4^th^ day of illness, the expression levels of IL-1β, IFN-γ, and IL-12α induced by genotype VIII were lower than those induced by the type VII strain [44]. The current study evaluated the difference in clinical pathogenic characteristics associated with an NDV virulent genotype VII GM strain, which has caused significant detriments in South China, compared to the attenuated vaccine strain La Sota. Study chickens expressed symptoms, such as lack of energy, appetite ablation, and mouth breathing following GM infection. Moreover, IL-1β was released in large quantities; subsequently, the animals died within 6 days with 100% mortality. However, this was not the case with the attenuated strain, as a few chickens expressed mild clinical symptoms on the first day, followed by gradual recovery. Furthermore, only minute amounts of IL-1β were detected in the organs of these chickens. These results are consistent with those of other published studies that describe the characteristics of NDV and have confirmed the importance of the release of pro-inflammatory cytokines, such as IL-1β, induced by the NDV virulent strain in the deadly immune response to this disease in chickens.

IL-1β is the central mediator of the inflammatory response, and neutralizing IL-1β using a specific antibody has been shown to reduce the inflammatory response to influenza A virus [8]. Studies have shown that following infection with NDV virulent strains, various organs exhibited varying degrees of exudative inflammation and inflammatory factors, such as IL-1β, IL-6, IL-18 and IFN-β, which were released in large quantities, resulted in high animal mortality rates, and were also observed in Experiment 1 of this study [1, 2, 45]. Alternatively, infection with lentogenic strains resulted in only mild non-life-threatening clinical symptoms, with all chickens eventually recovering. However, replacing specific lentogenic strain genes with those of the virulent strain followed by administration to SPF chickens induced severe illness. Furthermore, IL-1β expression increased significantly, as did the degree of inflammatory damage to the organs, leading to mortality [46]. These results indicated that virulent NDV induces IL-1β expression as well as massive accumulation of inflammatory cells, which exacerbated body organ damage and increased animal mortality. We also showed that NDV induced IL-1β expression in vitro and in vivo. Specifically, IL-1β was highly expressed in the lungs and glandular stomach, which are important target organs of NDV, in chickens infected with the virulent strain compared to those infected with the attenuated virus. Moreover, when infected at a MOI of 1, GM NDV induced higher levels of IL-1β than those induced by the La Sota virus in a time-dependent manner, which is consistent with the results of previous studies [47]. Furthermore, as previously noted, treatment of chickens with anti-IL-1β antibodies following infection with GM NDV decreased IL-1β level in organs, reduced body temperature and decreased the mortality rate, further confirming an essential role for IL-1β in the virulence of NDV infection.

The NLRP3 inflammasome is a key player in the maturation and production of IL-1β. This inflammasome is primarily composed of the NLRP3 scaffold, ASC, and caspase-1. Among them, the NLRP3 scaffold serves to connect ASC and caspase-1. Caspase-1 can then cleave the IL-1β precursor to form mature IL-1β; therefore, NLRP3/caspase-1 have vital roles in the production of IL-1β. Furthermore, previous studies have shown that NDV replication is required for NLRP3 inflammasome activation, and the secretion of IL-1β induced by the NDV strain Herts/33 decreased following NLRP3 knockdown via small hairpin RNA [4]. Thus, we sought to examine the ability of NDV to activate NLRP3 in chickens and other avian cells. The results showed that NDV activated NLRP3 and caspase-1 in vivo and in vitro. Additionally, following overexpression of NLRP3, IL-1β expression increased. In contrast, inhibition of NLRP3 or caspase-1 caused a significant decrease in IL-1β expression, indicating that the NLRP3/caspase-1 axis was involved in NDV-induced IL-1β expression in avian cells, which is consistent with experimental results in mammals [4].

Furthermore, we demonstrated that UV-inactivated NDV failed to induce IL-1β expression, suggesting that the replication of NDV is necessary for IL-1β activation. Various viral components produced by the virus during replication can induce IL-1β expression, including viral nucleic acids, ion channel proteins, and non-structural proteins. For example, genomic RNA and the M2 and PB1-F2 proteins of influenza virus have been shown to activate NLRP3 inflammasomes and promote the maturation of IL-1β [5, 32]. Similarly, the 2B protein of encephalomyocarditis virus decreases calcium ion concentrations in the Golgi apparatus in mouse bone marrow-derived macrophages, thereby promoting the secretion of IL-1β [34]. In another example, the non-structural protein 3D of enterovirus 71 directly activates NLRP3 inflammatory bodies to induce IL-1β expression [31]. Hence, to further identify the viral components involved in NDV-induced IL-1β, we obtained GM NDV from viral-infected cell culture supernatants via ultracentrifugation. Viral RNA was extracted and transfected into cells. As the transfection time increased, the expression of IL-1β induced by GM NDV RNA increased gradually, and the IL-1β protein concentration was found to be significantly higher than that in the control group, indicating that GM RNA induced IL-1β expression. Following overexpression of NLRP3, IL-1β expression induced by GM NDV RNA increased. Alternatively, inhibition of NLRP3 or caspase-1 significantly decreased IL-1β expression. Hence, this is the first study, to our knowledge, to demonstrate that GM NDV RNA induces IL-1β expression, which is dependent on NLRP3/caspase-1 activation. However, many questions remain to be answered. For example, it is unclear which components of NDV viral RNA (V genomic RNA, antigenomic RNA, or messenger RNA [48]) are required for the induction of IL-1β. Furthermore, since NDV is a *paramyxovirus*, the role, if any, of the non-structural V and W proteins in IL-1β activation must be investigated because during the process of paramyxovirus infection, non-structural proteins play a regulatory role in the inflammatory response. For example, Sendai virus V protein inhibits IL-1β secretion by preventing NLRP3 inflammasome assembly [49]. Similarly, the HPIV3 C protein interacts with NLRP3 to trigger proteasomal degradation of the NLRP3 protein and antagonize IL-1β expression [50]. Alternatively, the loss of Nipah virus C protein expression induces increased expression of IL-1β [51].

This study found that anti-IL-1β neutralizing antibody treatment significantly decreased the mortality rate from 100% to 71% in a lab-controlled virulent ND infection model. Furthermore, this study found that GM RNA induced IL-1β expression and activation of NLRP3/caspase-1. Ultimately, this study serves to clarify the importance of IL-1β in the pathogenesis of NDV infection, as well as to elucidate the molecular mechanisms involved with IL-1β expression induced by NDV infection.

## Supplementary information

**Additional file 1:** Specific detection of chicken IL-1β neutralizing antibody by Western blot. M: protein molecular weight standard; 1: pCAGGS empty vector, 2: chicken IL-1β protein, IL-1β neutralizing antibody was used as the primary antibody.
