# Supplementary figures and images for: Newcastle disease virus RNA-induced IL-1β expression via the NLRP3/caspase-1 inflammasome
Source: Vet Res. 2020 Apr 10;51:53. doi: 10.1186/s13567-020-00774-0 (PMC7156904; doi:10.1186/s13567-020-00774-0)

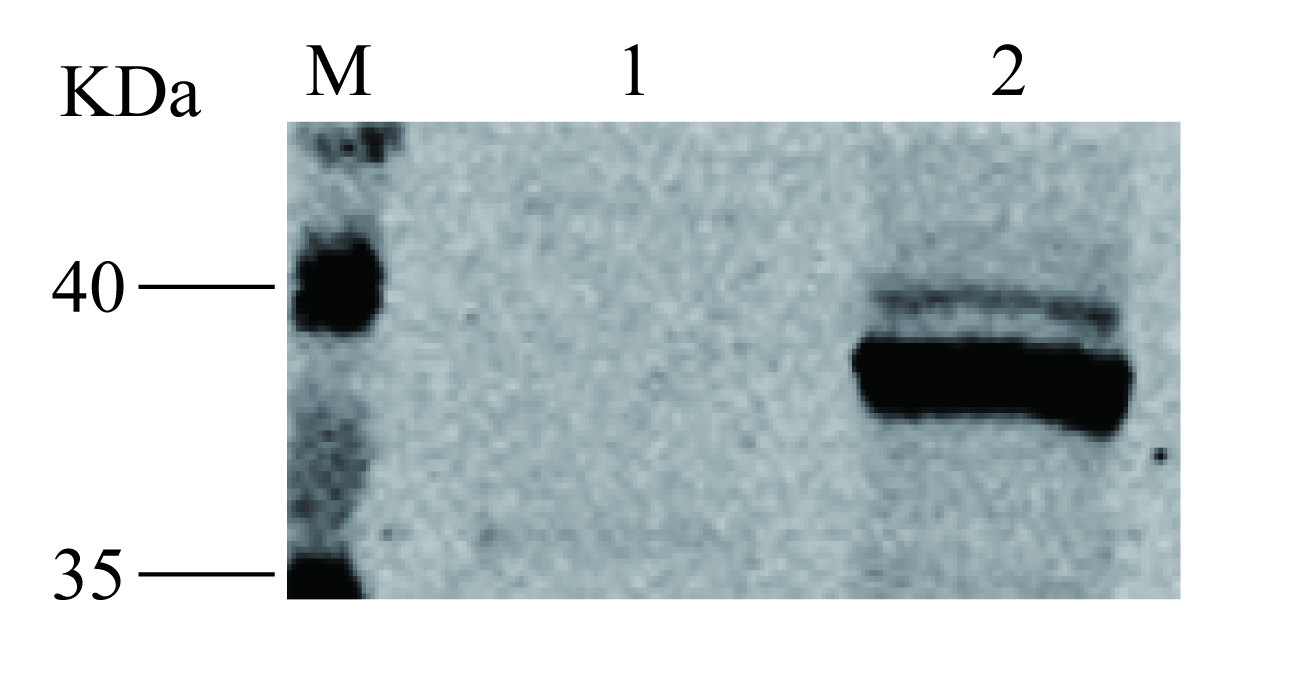

Supplement: Supplementary file 1 — Additional file 1: Specific detection of chicken IL-1β neutralizing antibody by Western blot. M: protein molecular weight standard; 1: pCAGGS empty vector, 2: chicken IL-1β protein, IL-1β neutralizing antibody was used as the primary antibody. [file 13567_2020_774_MOESM1_ESM.tif]
